# Cannabidiol reduces synaptic strength and neuronal firing in layer V pyramidal neurons of the human cortex with drug-resistant epilepsy

**DOI:** 10.3389/fphar.2025.1627465

**Published:** 2025-07-22

**Authors:** Vladimir A. Martinez-Rojas, Luis A. Márquez, Christopher Martinez-Aguirre, Isabel Sollozo-Dupont, Félix Iván López Preza, Monserrat Fuentes Mejía, Mario Alonso, Luisa Rocha, Emilio J. Galván

**Affiliations:** ^1^ Departamento de Farmacobiología, Cinvestav Sur, Mexico City, Mexico; ^2^ Dominick P. Purpura Department of Neuroscience, Albert Einstein College of Medicine, Bronx, NY, United States; ^3^ Instituto Nacional de Perinatología, Isidro Espinosa de los Reyes, Mexico City, Mexico; ^4^ International Center for Epilepsy Surgery, HMG-Coyoacán Hospital, Mexico City, Mexico; ^5^ Centro de Investigaciones sobre el Envejecimiento, CIE, Mexico City, Mexico

**Keywords:** cannabidiol, human cortex, layer V pyramidal neuron, patch-clamp recordings, ion channels

## Abstract

The use of cannabidiol (CBD) as an alternative pharmacological approach for the symptomatic management of epilepsy has gained attention due to its potential efficacy, particularly in drug-resistant cases of epilepsy. Although multiple studies have described that CBD reduces neuronal hyperexcitability, the mechanistic basis of CBD remains a topic of ongoing research. In this study, we provide an electrophysiological portrayal of CBD’s effects on the glutamatergic transmission and intrinsic excitability of layer V pyramidal neurons of the human neocortex resected from drug-resistant epilepsy patients. The perfusion of CBD transiently depressed the field excitatory potential amplitude elicited in layer I/II and recorded in layer V without altering the paired-pulse ratio, suggesting a postsynaptic locus of action for CBD. Cortical slices perfused with 4-aminopyridine exhibited an increased number of spontaneous synaptic events that were abolished in the presence of CBD. At the cellular level, whole-cell patch-clamp recordings showed that CBD decreased the excitability of layer V pyramidal neurons, as evidenced by changes in the somatic input resistance, the membrane time constant, the hyperpolarization-induced “sag” conductance, the rheobase current needed to elicit an action potential, and the firing discharge in response to depolarizing current steps. Consistent with the last observation, CBD decreased the amplitude of the TTX-sensitive inward currents without altering the kinetics of the macroscopic outwardly directed currents. CBD washout restored the passive and active electrophysiological properties of pyramidal neurons. Collectively, these experiments demonstrate that CBD decreases the neuronal excitability of human cortical neurons from patients with drug-resistant epilepsy.

## Introduction

Epilepsy is a neurological disorder that involves atypical electrical activity and the occurrence of spontaneous recurrent seizures (Fisher et al., 2014). Anti-seizure medications (ASMs) are the first line of drug therapy for reducing neuronal excitability and seizure frequency (Malekmohammad et al., 2024). Despite the advances in ASMs, around one-third of epilepsy patients exhibit drug resistance to conventional ASMs (Gonzalez-Giraldo and Sullivan, 2020; Löscher et al., 2020). Therefore, drug-resistant epilepsy (DRE) is observed when two well-chosen and well-tolerated antiepileptic drug treatments fail to reduce seizure frequency (Kwan et al., 2010). This phenomenon underscores the critical issue of drug resistance, which remains a significant obstacle in epilepsy therapy.

Within this framework, cannabidiol (CBD), a non-euphoric compound derived from the *Cannabis* plant, is a drug approved by multiple regulatory agencies for treating drug-resistant seizures associated with epileptic encephalopathies, such as tuberous sclerosis complex and the Dravet and Lennox–Gastaut syndromes (Rosenberg et al., 2017; von Wrede et al., 2021). While CBD’s clinical efficacy in DRE has been reported (Devinsky et al., 2018), the underlying neuronal mechanisms remain largely unknown. Previous studies have demonstrated that CBD is a functional agonist or antagonist of ion channels, neurotransmitter receptors, transporters, and enzymes (Ibeas Bih et al., 2015; Franco et al., 2019). In the hippocampus, a critical region for neural hyperexcitability and a primary driver of temporal lobe seizures, data from animal models indicate that CBD exerts both antiepileptic and neuroprotective effects (Khan et al., 2018; Lima et al., 2020). For instance, in a model of chronic lithium–pilocarpine-induced epileptogenesis, CBD restores the excitatory/inhibitory ratio and decreases the excitability level of the hippocampus (Rosenberg et al., 2023). In another study using multielectrode array recordings, CBD attenuated the bursting activity of CA1 neurons elicited by 4-aminopyridine (4-AP; Jones et al., 2010). Comparable effects have been reported in the neocortex, as CBD reduced seizure-like events and inter-ictal bursting (Javadzadeh et al., 2024). The suppression of pathological network activity exerted by CBD aligns with its demonstrated actions on the voltage-gated Na^+^ channels. In this sense, Mechanistic insights from expression systems data show that CBD inhibits Na^+^ channels through two distinct binding sites, providing a compelling framework for the modulation of synaptic transmission and intrinsic excitability in intact brain tissue (Sait et al., 2020; Huang et al., 2023).

Given CBD’s efficacy in restraining seizure activity in different animal models, this study aimed to determine the effect of CBD on the synaptic transmission and intrinsic excitability of pyramidal neurons located in layer V of the human neocortex and resected from DRE patients. Collectively, our results demonstrate that CBD transiently depresses glutamatergic transmission and decreases the overall somatic excitability of pyramidal neurons.

## Results

### CBD modulates the synaptic transmission of the human cortex

Electrophysiological recordings aimed at examining the neurophysiological effects of CBD were performed in acute cortical slices obtained from six samples of brain tissue resected from the temporal (n = 3) and the frontal (n = 3) cortex of adult patients (21 to 30 years old) diagnosed with DRE (Table 1 shows the clinical data for each patient). Some of the brain tissue used in this study has been described in previous publications (Martínez-Rojas et al., 2023; Martínez-Aguirre et al., 2023).

**TABLE 1:** Clinical data of neocortical tissue resected from patients with drug-resistant epilepsy.

Surgery date	Sex	Age (years)	Seizure onset (years)	Duration (years)	Seizure frequency (per month)	Location of epileptic focus
July, 22	Female	21	10	11	7	Temporal neocortex
August, 22	Male	21	13	8	12	Temporal neocortex
July, 22	Female	30	13	17	20	Frontal neocortex
January, 23	Female	27	NA	NA	NA	Temporal neocortex
March, 23	Male	16	10	6	150	Frontal neocortex
May, 23	Female	24	16	8	NA	Frontal neocortex

**FIGURE 1:**
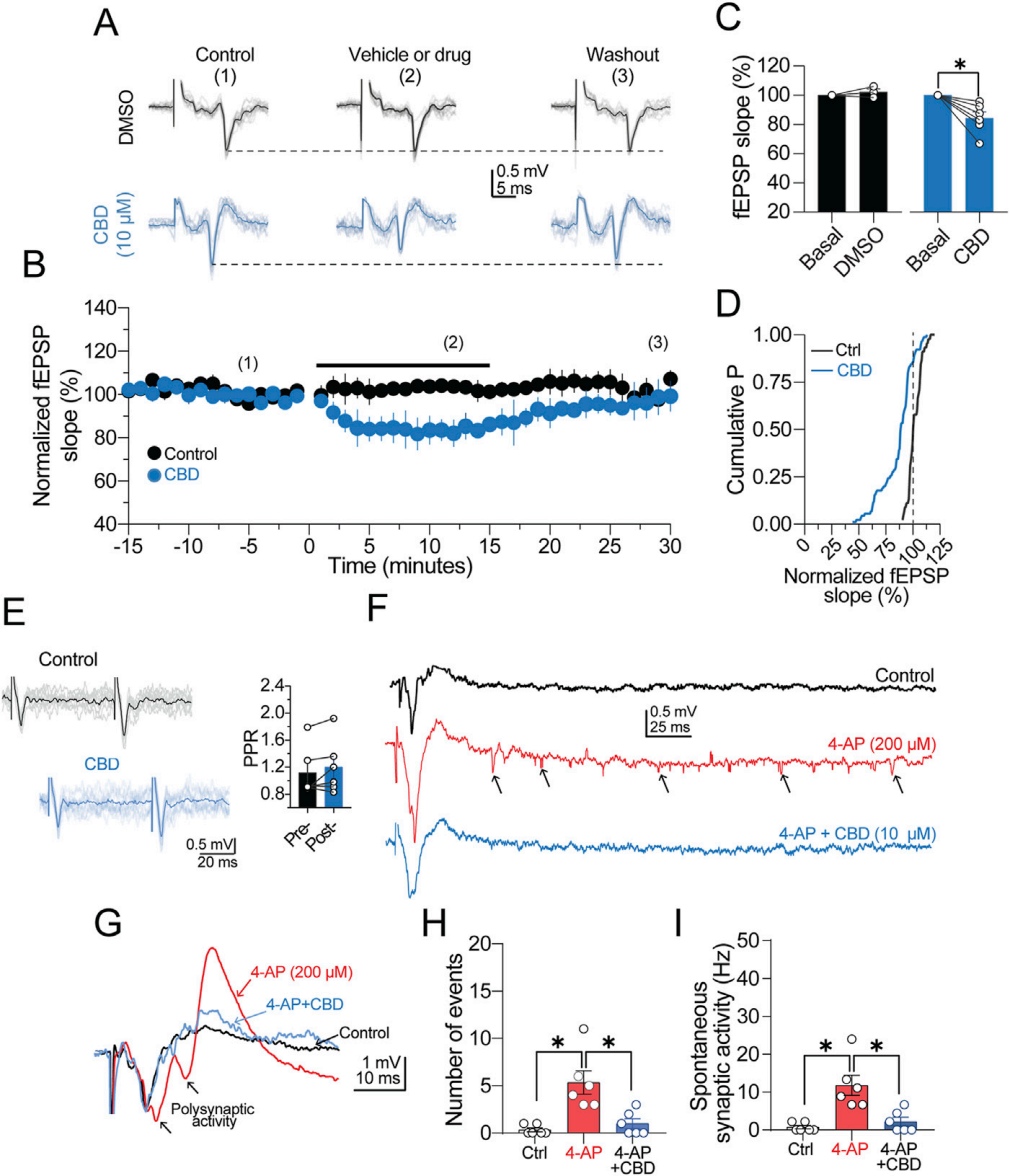
CBD depresses synaptic transmission. **(A)** Representative traces of field excitatory postsynaptic potentials (fEPSPs) recorded from human neocortical slices. In the middle panel, vehicle or drug condition refers to perfusion of DMSO or DMSO + CBD. For conditions 2 and 3, the synaptic responses were collected at the end of drug perfusion or 30 min after the washout, respectively. **(B)** Time-course graph of normalized fEPSP slopes. The black circles are the fEPSPs in the presence of DMSO, and the blue circles show the depressing effect of CBD on synaptic transmission. **(C)** Bar graph summarizing the effect of DMSO or CBD on the fEPSP slope. **(D)** Cumulative distribution chart showing the fEPSP slope distribution in the control condition or presence of CBD. The minimal variation observed in the normalized fEPSP slope of the control response (see vertical dashed line for reference) indicates a stable synaptic response. **(E)** Over imposed traces of paired-pulse responses (acquired with 60 ms inter-stimulus interval). Right panel, bar graphs showing the lack of changes in the paired-pulse ratio in the presence of CBD. **(F)** Representative traces showing an evoked fEPSP and the spontaneous synaptic activity observed in control condition (ACSF, black trace), in the presence of 4-AP (red trace; 200 μM), and 4-AP + CBD (blue trace). Notice the 4-AP-induced increase in spontaneous synaptic activity (arrowheads) and its suppression in the presence of CBD. **(G)** Representative traces showing the an evoked fEPSP, the resulting polysynaptic activity induced by 4-AP (red trace) and the suppression of the exacerbated response in the presence of CBD (blue trace). **(H)** Barg graph quantifying the number of spontaneous synaptic events in the three experimental conditions. **(I)** Comparison of the frequency of spontaneous synaptic activity in panel **(E)** n = 6; *p < 0.05; Student’s paired t-test.

First, we performed extracellular recordings to determine the effect of CBD on synaptic transmission. A bipolar electrode was placed in LI/II, and a recording pipette was positioned in layer V. From a series of exploratory experiments (n = 3 slices from the temporal cortex/2 patients), we determined that synaptic responses could persist for up to 2 h without exhibiting changes in the kinetics of the field excitatory postsynaptic potential (fEPSP). Figure 1A shows a series of over imposed fEPSPs and the average response obtained under our experimental conditions. Next, a baseline response of fEPSPs acquired with a paired-pulse protocol (60-ms inter-stimuli interval; see also representative traces in Figure 1E) was recorded for 15 min, followed by CBD perfusion (10 μM, 15 min) and subsequent washout (n = 3 patients for the temporal cortex; n = 2 patients for the frontal cortex). As illustrated in the time-course graph in Figure 1B, CBD caused a depression of the fEPSP slope of ≈16% (84.3% ± 4.2% of baseline; p < 0.05, Wilcoxon test; n = 6 slices/5 patients; 100.0% responsive slices; Figure 1C), and the synaptic response returned to baseline following washout. To discard that the depressive effect of CBD may result from Dimethyl sulfoxide (DMSO) (0.05%), which was used as the CBD vehicle, we also examined the effect of DMSO on the fEPSPs acquired from the temporal (n = 1 patient) and the parietal (n = 2 patients) cortex. As expected, the depressive effect of CBD was vehicle independent, as DMSO did not modify the fEPSP slope (102.2% ± 2.2% of the baseline response; p > 0.05, Wilcoxon test; n = 3 slices/3 patients; Figures 1A,C, black symbols). Interestingly, the paired-pulse ratio (PPR) of the fEPSP did not exhibit changes during CBD perfusion (PPR in the control condition = 1.1 ± 0.1; PPR in the presence of CBD = 1.2 ± 0.2; ns; Figure 1E), suggesting a postsynaptic locus of action of CBD. A probability distribution analysis constructed from all the synaptic responses during CBD or vehicle perfusion corroborated that the synaptic responses were mostly decreased by CBD (Figure 1D).

Next, we evaluated the effects of CBD on the spontaneous synaptic activity induced by the K^+^ channel blocker 4-AP (200 μM; n = 1 temporal cortex slice and 2 frontal cortex slices; Figure 1F). Perfusion of 4-AP triggered mild polysynaptic activity in fEPSPs (Figure 1F, black arrowheads in the red trace), followed by an increase in the number of synaptic events (Figure 1E, black arrows in the red trace), which were considered spontaneous synaptic activity. Under these experimental conditions, perfusion of CBD not only suppressed polysynaptic activity (Figure 1F, blue trace) but also decreased both the number (4-AP = 5.3 ± 1.2; 4-AP + CBD = 1.0 ± 0.5; p < 0.05, Friedman test; 100.0% responsive slices; Figure 1H) and the frequency of spontaneous events (4-AP = 11.8 ± 2.7 Hz; 4-AP + CBD = 2.2 ± 1.6 Hz; p < 0.05, Friedman test; n = 6 slices/3 patients; 100.0% responsive slices; Figure 1I). The reduction the in the spontaneous synaptic activity elicited by CBD in the presence of 4-AP is shown in the traces in Figure 1G. Together, these results show that CBD decreases the strength of synaptic transmission and spontaneous synaptic activity in cortical tissue from DRE patients.

### CBD decreases the intrinsic excitability of human pyramidal neurons

**FIGURE 2:**
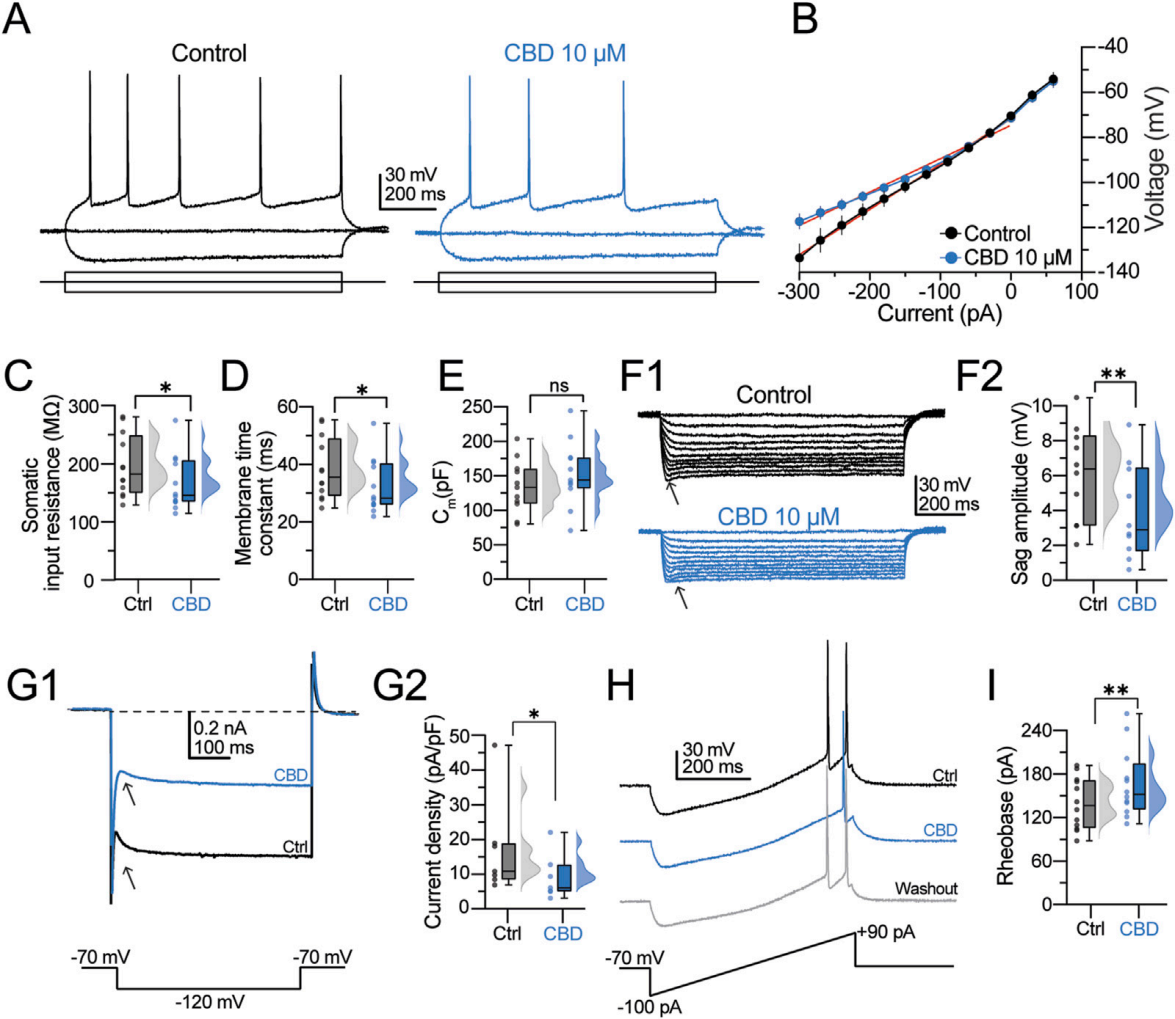
CBD modifies the membrane properties and intrinsic excitability of pyramidal neurons from the human neocortical neurons. **(A)** Representative voltage traces at a holding potential of −70 mV in response to a 1-s current injection (−90 and +90 pA) in the control condition and presence of CBD. **(B)** I–V scatter plot. CBD increases the inward rectification. **(C–E)** Raincloud plots contrasting the effects of CBD on the passive membrane values. The shaded dots in the plots represent individual cells. (**F1)** Voltage responses evoked with a hyperpolarizing current injection. Arrowheads indicate the hyperpolarization-induced sag deflection. **(F2)** Raincloud plot contrasting the sag values in both experimental conditions. **(G1)** Representative trace of the hyperpolarization-activated *I*_h_ current. **(G2)** Raincloud plot contrasting the current densities in the control condition and after CBD perfusion. **(H)** Representative voltage ramp traces contrasting the latency and current necessary to elicit an action potential. CBD increases the latency to fire a single AP and the rheobase current. **(I)** Raincloud plot showing the increased rheobase current in the presence of CBD. n = 12; *p < 0.05; **p < 0.01; Student’s paired t-test.

For the next experiments, we switched to whole-cell patch-clamp recordings. Under differential interference contrast (DIC) microscopy, pyramidal neurons were somatically located in layer V of the neocortex, 50–150 µm below the slice surface. Consistently, the neurons in this study exhibited the typical pyramidal cell morphology, including a broad base and a pointed apex with the major dendritic branch pointing toward the cortex-pia. After the initial giga seal break-in of the whole-cell configuration, pyramidal neurons were allowed to stabilize for 5 min before switching to current-clamp mode. Under this configuration, the neurons exhibited a hyperpolarized resting membrane potential (RMP; −68.19 ± 1.54 mV; n = 22 neurons/6 patients) and barely showed spontaneous synaptic activity. Neurons with a depolarized membrane potential (above −55 mV) or increased spontaneous activity were excluded from the analysis (n = 8 neurons/3 patients). Our previous studies found that the predominant firing pattern of pyramidal neurons is regular spiking (Martínez-Aguirre et al., 2023; Martínez-Rojas et al., 2023). For this study, we restricted our experiments to regular spiking cells. To identify these cells in the acute slice, neurons were held in current-clamp mode at −70 mV, and a hyperpolarizing or depolarizing pulse (−90/+90 pA, 1 s) was somatically injected. Figure 2A, left panel, shows the typical regular firing discharge with a marked afterhyperpolarization phase and increased adaptation, whereas the right panel shows a decreased firing discharge in the presence of CBD (10 µM). Figure 2B shows an average I–V scatter plot in the control condition (black symbols) and CBD (blue symbols). CBD did not alter the RMP (−70.43 ± 1.88 mV; n = 12 neurons/6 patients; t-test: t_(11)_ = 1.1, p = 0.3). However, it is noticeable in the I–V plot that CBD increases the inward rectification in the hyperpolarizing range. Consistent with this finding, CBD decreased the somatic input resistance (*R*_N_) (*R*_N_ in the control condition: 199.9 ± 12.6 MΩ; in the presence of CBD: 175.1 ± 11.3 MΩ; t-test: t_(11)_ = 2.4, p = 0.03; n = 12 neurons/6 patients; 66.7% responsive neurons; Figure 2C) and increased the membrane time constant (τ_memb_) (τ_memb_ in the control condition: 30.9 ± 3.4 ms; in the presence of CBD: 33.7 ± 3.4 ms; t-test: t_(11)_ = 2.5, p = 0.02; n = 12 neurons/6 patients; 75.0% responsive neurons; Figure 2D) without altering the membrane capacitance (*C*_m_) of the recorded neurons (*C*_m_ in the control condition: 143.0 ± 9.6 pF; in the presence of CBD: 158.6 ± 11.9 pF; t-test: t_(11)_ = 1.1, p = 0.3; n = 12 neurons/6 patients; Figure 2E). From the hyperpolarizing traces of the I–V curve, we also found that CBD decreases the “sag” amplitude (Figure 2F1, arrowheads) of layer V pyramidal neurons (sag amplitude in the control condition = 6.2 ± 0.7 mV; in the presence of CBD = 4.3 ± 0.7 mV; t-test: t_(11)_ = 4.1, p = 0.002; n = 12 neurons/6 patients; 90.0% responsive neurons; Figures 2F1, 2F2). Since the voltage sag is mediated by the hyperpolarization-activated current (*I*_h_), we further investigated the effect of CBD on the current density of *I*_h_ with voltage-clamp recordings. Pyramidal neurons were held at −70 mV, and a hyperpolarizing step to −120 mV was applied (Figure 2G1, bottom panel). Compared with the baseline response (Figure 2G1, black traces and arrowheads), CBD decreased the hyperpolarization-activated *I*_h_ current (*I*_h_ in the control condition: 15.0 ± 4.3 pA; in the presence of CBD = 8.5 ± 2.0 pA; t-test: t_(6)_ = 2.8, p = 0.03; n = 7 neurons/3 patients; 100.0% responsive neurons; Figures 2G1, 2G2). Next, we measured the rheobase current necessary to elicit one action potential (AP). For this, we injected a current ramp (from −100 to +90 pA within 500 ms, dI/dt = 0.38 pA/ms; Figure 2H, bottom panel) and determined the current and latency for the firing response. As illustrated in the representative traces in Figure 2I, CBD increased the rheobase current needed to evoke a single action potential (rheobase current in the control condition = 98.2 ± 10.2 pA; in the presence of CBD = 115.7 ± 12.2 pA; t-test: t_(11)_ = 4.1, p = 0.001; n = 12 neurons/6 patients; 91.6% responsive neurons; Figures 2H,I) and increased the firing latency (action potential latency in the control condition = 142.2 ± 12.2 ms; in the presence of CBD = 173.9 ± 16.8 ms; latency after washout = 150.06 ± 20.45 ms; t-test: t_(11)_ = 2.7, p = 0.01; n = 12 neurons/6 patients). These experiments indicate that CBD controls the passive and active electrophysiological properties of pyramidal neurons to reduce the excitability level of layer V human pyramidal neurons.

### CBD modifies the spike dynamics of human neocortical neurons

**FIGURE 3:**
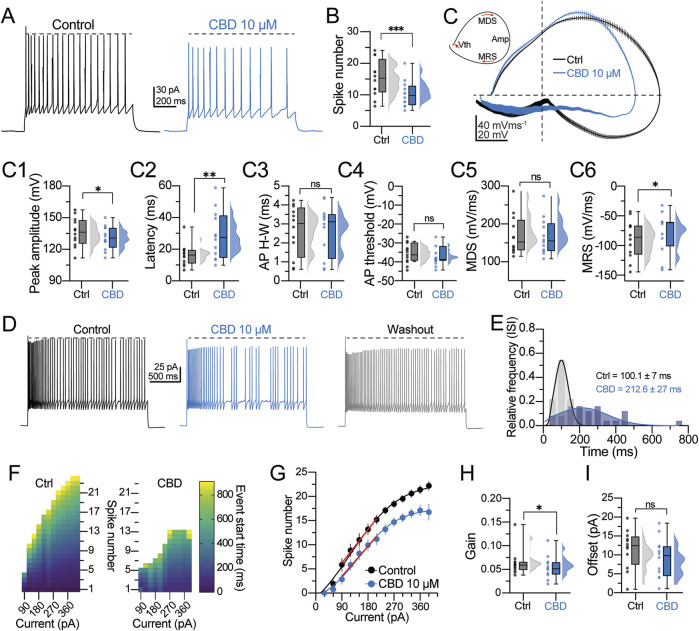
CBD modifies the action potential spike kinetics and firing properties of pyramidal cells. **(A)** Representative voltage traces of a regular spiking neuron and the response to CBD. The horizontal dashed line indicates the spike amplitude reduction observed in the presence of CBD. **(B)** Raincloud plot showing the decreased number of action potentials in response to CBD. **(C)** Upper left inset: schematic representation of a phase plot extracted from an action potential (AP) spike. *V*th is the voltage threshold for AP spike initiation; MDS is the maximal depolarization slope of the AP spike; Amp is the peak amplitude of the AP spike; MRS is the maximal repolarization slope of the AP spike. Right panel: averaged phase plots obtained from the data in **(A)**, showing the dynamic progression of the membrane potential during the AP spike in both experimental conditions. Notice the decreased AP amplitude in the presence of CBD. **(C1−C6)** Raincloud plots summarizing the changes in the AP spike waveform kinetics. Data for these analyses were extracted from the phase plot analysis. **(D)** Voltage traces showing the firing discharge in response to a 5-s depolarization in the control condition, CBD, and washout. Notice the jitters in the membrane potential and the reduced firing frequency in the presence of CBD. **(E)** Histogram of the relative frequency distribution of the mean inter-spike interval (ISI) computed from the neurons assessed in **(D)**. **(F)** Heatmaps showing the output response to current injection (from −90–360 pA, Δ30 pA) in a representative neuron before and after CBD perfusion. The color coding represents the start time of each spike event during the stimulation period. **(G)** Spike numbers plotted as a function of the current injection fitted to a sigmoid function to compute the **(H)** neuronal output gain in the control condition and the presence of CBD or **(I)** fitted to a linear function to compute the neuronal offset. n = 12; *p < 0.05; **p < 0.01; ***p < 0.001; Student’s paired t-test.

Next, the effects of CBD on the firing discharge and single AP dynamics were determined. Neurons were held at −70 mV, and the firing discharge was evoked with a depolarizing current step (200 pA, 1- s duration). Figure 3A shows the typical firing discharge evoked in a regular spiking neuron, whereas the right panel shows the effect of CBD (10 μM, blue traces). CBD reduces the action potential firing frequency (spike frequency in the control condition = 15.5 ± 1.2 Hz; in the presence of CBD = 11.8 ± 0.9 Hz; t-test: t_(11)_ = 3.7, p = 0.003; 12 neurons/6 patients; 83.3% responsive neurons; Figure 3B). From the voltage traces in Figure 3A, it is apparent that CBD modifies the action potential amplitude. Therefore, we analyzed the first derivative of the AP waveform (dV/dt) to determine the AP spike’s kinetic components. Figure 3B shows the phase plots obtained from a representative neuron in the control condition (black line) and in the presence of CBD (blue line). Bath perfusion of CBD caused a decrease in the spike peak amplitude (peak amplitude in the control condition = 135.5 ± 3.4 mV; in the presence of CBD = 130.9 ± 2.8 mV; t-test: t_(11)_ = 2.2, p = 0.04; 66.6% responsive neurons; Figure 3C1) and increased the latency to the spike interval (latency in the control condition = 17.1 ± 1.9 ms; in the presence of CBD = 28.7 ± 4.1 ms; t-test: t_(11)_ = 3.2, p = 0.008; 100.0% responsive neurons; Figure 3C2). No statistical difference was found in the spike half-width (H-W) (H-W in the control condition = 2.8 ± 0.3 ms; in the presence of CBD = 2.8 ± 0.4 mV; t-test: t_(11)_ = 0.22, p = 0.8; Figure 3C3) and in the voltage threshold to fire an action potential (threshold in the control condition = −36.3 ± 1.4 mV; in the presence of CBD = −38.3 ± 1.2 mV; t-test: t_(11)_ = 2.1, p = 0.06; Figure 3C4). Likewise, CBD perfusion did not alter the maximum depolarizing slope (MDS) (MDS in the control condition = 168.8 ± 14.4 mV/ms; in the presence of CBD = 166.2 ± 13.2 mV/ms; t-test: t_(11)_ = 0.9, p = 0.4; Figure 3C5) but decreased the maximum repolarizing slope (MRS) of the pyramidal neurons’ spikes (MRS in the control condition = −86.0 ± 8.2 mV/ms; in the presence of CBD = −76.2 ± 8.7 mV/ms; t-test: t_(11)_ = 2.4, p = 0.03; 83.3% responsive neurons; Figure 3C6). Next, we evaluated the firing response during a sustained depolarizing step (200 pA/5 s) and its response to CBD. In the control condition, pyramidal neurons exhibited a regular firing discharge that reached a maximal firing frequency of 11.21 ± 1.2 Hz (Figure 3D, left panel). In the presence of CBD, the firing discharge became irregular, with silent periods and clusters of spikes that reduced the firing frequency (7.8 ± 1.5 Hz; t-test: t_(5)_ = 3.0, p = 0.02; n = 6 neurons/3 patients; Figure 3D, middle panel). Although CBD washout partially restored the firing frequency (Figure 3D, right panel), the action potential amplitude exhibited an amplitude reduction (see horizontal dashed lines). Due to the firing response changes, we also analyzed the inter-spike intervals (ISIs) (Martínez-Aguirre et al., 2023). Compared with control cells, CBD increased the mean ISI duration (ISI in the control condition = 100.1 ± 7.0 ms; in the presence of CBD = 212.6 ± 26.9 ms; 75.0% responsive neurons; Figure 3E). The increased ISI in the presence of CBD indicates a decreased firing rate. A quantification of the number of spikes as a function of increasing current amplitude, ranging from 0 to 360 pA (Δ30 pA), is depicted in Figure 3F. In the control condition, pyramidal neurons exhibited a consistent and progressive increase in spike frequency in response to the current amplitude increase. By contrast, bath perfusion of CBD reduced the number of spikes across all current steps, corroborating its inhibitory effect on neuronal output (Figure 3F). The scatter plot in Figure 3G shows the number of spikes as a function of injected current. The data obtained from this graph were fitted to a sigmoidal function and a linear regression to determine the neuronal gain and offset parameters, respectively. Consistent with the previous experiments, CBD caused a significant decrease in the neuronal gain (neuronal gain in the control condition = 0.064 ± 0.008 Hz/pA; in the presence of CBD = 0.053 ± 0.006 Hz/pA; t-test: t_(11)_ = 2.5, p = 0.03; 75.0% responsive neurons; Figure 3H) with no substantial change in the offset (offset in the control condition = 11.4 ± 1.5 pA; in the presence of CBD = 9.1 ± 1.5 pA; t-test: t_(11)_ = 1.7, p = 0.12; Figure 3I). These findings suggest that CBD restrains the firing frequency and the temporal precision of spike generation in pyramidal neurons from DRE cortical tissue.

### CBD modulates the macroscopic currents of human neocortical neurons

**FIGURE 4:**
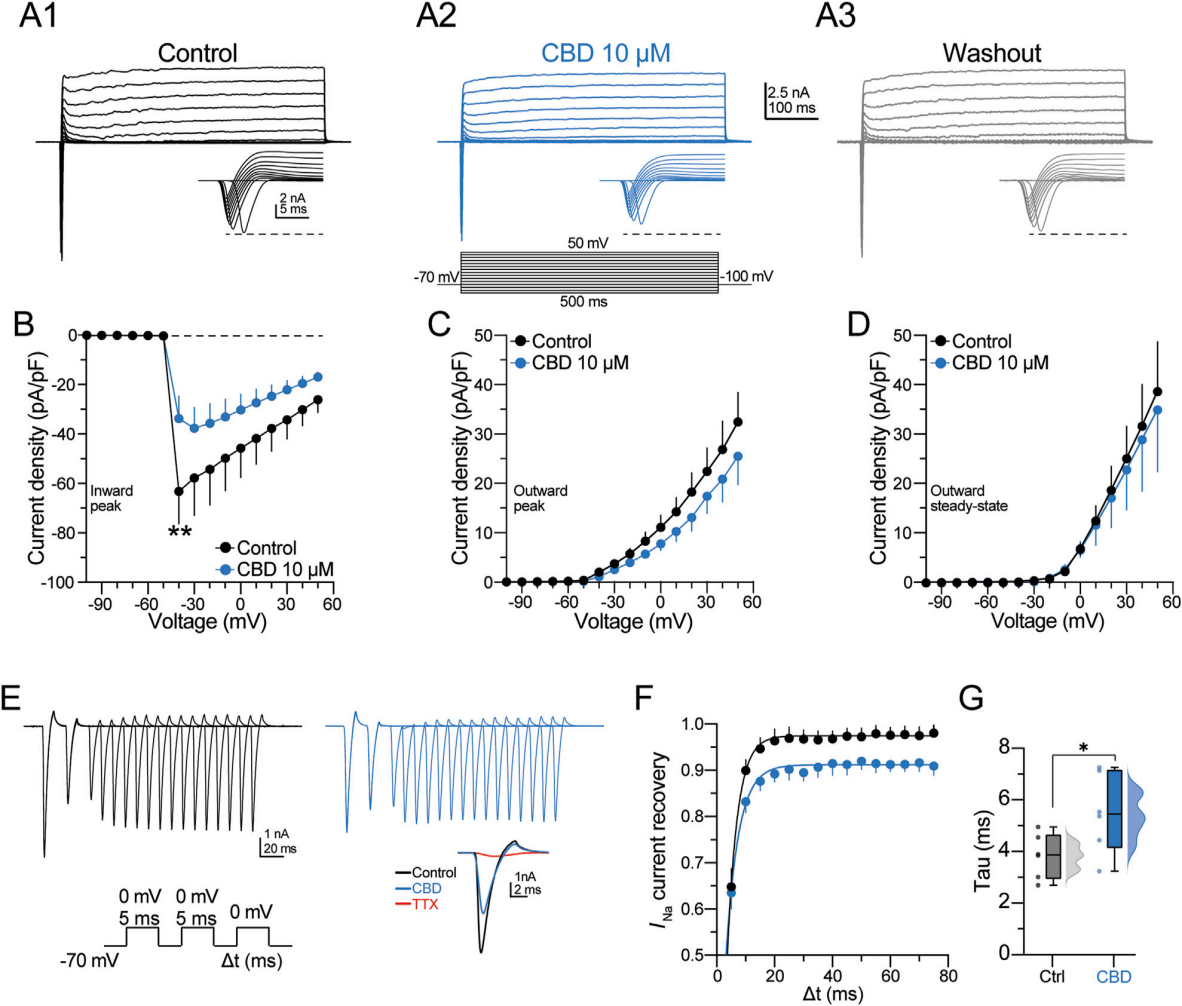
CBD blocks the voltage-activated sodium channels of pyramidal neurons. **(A)** Representative voltage-gated inward and outward macroscopic currents in the three experimental conditions. The bottom panel in **(A2)** shows the voltage protocol used to elicit the currents. The bottom right inset in each panel shows the fast-inactivating inward current; the horizontal dashed line contrasts the peak amplitude in the three experimental conditions. **(B)** Voltage–current (V–I) curve of the inward current deflection (peak) of the signal illustrated in **(A)**. **(C–D)** V–I curves of the outward current deflections measured at either the maximal peak or the steady state, respectively. CBD did not alter the kinetics of both components of the outward currents. **(E)** Somatically evoked fast-inactivating inward currents from a representative neuron. The current traces were obtained using the protocol in the bottom panel to determine the recovery time course from fast inactivation. The inset shows the pharmacological blockade of the inward current by TTX (1 μM, red trace). **(F)** Scatter plot representing the TTX-sensitive *I*_Na_ current recovery as a function of the interstimulus interval (Δt). Recoveries were fitted with one exponential, and **(G)** the constant recovery time (tau) values are displayed in the raincloud graph for both experimental conditions. n = 7; *p < 0.05; **p < 0.01; Student’s paired t-test.

The previous observations indicate that CBD selectively modulates the kinetic parameters of the AP waveform. Therefore, the next experiments were designed to determine the effect of CBD on the inward and outward macroscopic currents of pyramidal neurons. Currents were elicited by applying step voltage commands from −100 to +50 mV (500-ms duration; Figure 4A2, bottom panel), which elicited an inward current followed by an outward current (see current magnification in the bottom insets in Figures 4A1–4A3); then CBD (10 µM) was bath perfused, followed by washout (Figure 4A, middle and right panels). The V–I curve in Figure 4B summarizes the inhibitory effect of CBD on the inward current density of pyramidal neurons (inward current density at −40 mV in the control condition = −64.9 ± 16.5 pA/pF; in the presence of CBD = −32.6 ± 8.4 pA/pF; t-test: t_(5)_ = 2.6, p = 0.04; n = 6 neurons/3 patients; 100.0% responsive neurons). By contrast, CBD did not modulate the peak and steady-state outward currents (Figures 4C,D).

Next, a multiple voltage command protocol (a two-step protocol from −70 to 0 mV, lasting 5 ms, followed by a third pulse to 0 mV with an increasing interval of 5 ms [Δt]; Figure 4E, bottom panel) was used to determine the current recovery from fast inactivation. Figure 4E shows the currents elicited in the control condition and in the presence of CBD. At the end of the experiment, the fast-inactivating Na^+^ channel blocker tetrodotoxin (TTX, 100 nM) was bath perfused. TTX blocked ≈95% of the inward current (see red traces in the inset in Figure 4E). The scatter plot in Figure 4F shows that CBD slows the recovery from the inactivation state of the inward current, whereas the box plots in Figure 4G show that CBD increases the recovery time constant of the inward current (recovery time constant in the control condition = 3.7 ± 0.4 ms; in the presence of CBD = 5.4 ± 0.7 ms; t-test: t_(5)_ = 3.8, p < 0.01; n = 6 neurons/3 patients; 100.0% responsive neurons). These data suggest that CBD favors inhibition of the macroscopic Na^+^ currents and delays recovery from their inactivated state without modulating the outward potassium currents. Likewise, the data support the idea that CBD decreases the neuronal excitability of layer V pyramidal neurons of the DRE human neocortex via modulation of voltage-sensitive Na^+^ channels, as previously observed in animal models (Hill et al., 2014; Patel et al., 2016; Ghovanloo et al., 2019).

## Discussion

Multiple studies have confirmed the modulatory actions of CBD on neurotransmitter release, ion channel activity, and, more importantly, the reduction of hyperexcitability and epileptiform activity in *ex vivo*, *in vitro*, and *in vivo* models of epilepsy. Here, using extracellular and whole-cell patch-clamp recordings, we investigated the effects of CBD on synaptic transmission and intrinsic excitability in layer V human neurons obtained from resected tissue of patients with DRE. Our results corroborate findings from animal models and extend those findings to human tissue, showing that CBD transiently decreases the strength of synaptic transmission and reduces the frequency of 4-AP-evoked synaptic events. Likewise, the shift in passive membrane properties values (somatic input resistance, membrane time constant, and hyperpolarization-evoked sag conductance), in parallel with the increased rheobase current and latency to fire action potentials as assessed via patch-clamp recordings, corroborates the reduction in neuronal excitability consistently reported in animal models. To strengthen the last observation, we demonstrated that CBD decreases neuronal firing during prolonged periods of sustained depolarization. Lastly, we showed that CBD blocks the TTX-sensitive inward currents mediating *I*_Na_ without altering the kinetics of the outwardly directed K^+^ currents. Our findings strongly indicate that CBD decreases the excitability of layer V human neurons obtained from resected tissue of patients with DRE.

In a previous human tissue study, we reported that CBD reduced glutamate release from highly purified isolated nerve terminals (Martinez-Aguirre et al., 2023). We also identified 10 µM as the most effective concentration for suppressing glutamate release. We used the same CBD concentration in the present experiments based on those findings. Consistent with the dose of CBD chosen for this study, the same concentration of CBD causes transient inhibition of glutamatergic transmission and reduces the firing output of rat hippocampal neurons (Ledgerwood et al., 2011; Khan et al., 2018), supporting the notion that CBD dampens the hyperexcitability associated with experimental epilepsy.

CBD does not directly bind to the NMDAR or AMPAR receptors (Yu et al., 2020), suggesting an indirect mechanism for reducing the glutamatergic response. Possible mechanisms that may explain the transient depression observed in our study include, but are not limited to, CBD interacting with the cannabinoid CB1 receptor (McPartland et al., 2007), the adenosine A1 receptor (Carrier et al., 2006), the transient receptor potential vanilloid receptor (De Petrocellis et al., 2011), or the orphan G protein-coupled receptor, GPR55 (Ryberg et al., 2007). These receptors are strongly expressed in the human neocortex (Steffens et al., 2003; Mori et al., 2012; Klaft et al., 2016; García-Gutiérrez et al., 2018), and their interaction with CBD modulates the signaling cascades mediating glutamate release. Due to the inherent limitations in obtaining human tissue for our experiments, we did not explore those potential interactions. Future research must focus on exploring the interaction of CBD with those receptor systems in the human cortex beyond the DRE brain to confirm or discard their possible modulatory effects. Despite this limitation, our study provided solid experimental evidence of the effects of CBD on synaptic transmission in the human brain.

On the other hand, the patch-clamp experiments demonstrated a decrease in the values of passive membrane properties. For example, the augmented inward rectification in the hyperpolarized range of the I–V curve suggests a CBD-mediated increased gain-of-function of inwardly rectifying K^+^ channels, which promotes the hyperpolarized state and dampens somatic excitability. Interestingly, cannabinoid agonists can activate the inwardly rectifying K^+^ channels via CB1 receptors (Mackie et al., 1995); however, no information exists regarding CBD’s actions on the inwardly rectifying K^+^ currents. Similarly, our results suggest modulation of the large-conductance Ca^2+^-activated potassium (BK) channels, which are responsible for the fast repolarization of the action potential spike. BK channels control the action potential shape and regulate the afterhyperpolarization phase and the firing frequency of central neurons. Consistent with our observations, previous studies have reported modulation of BK channels by CBD in seizure models (Shirazi-Zand et al., 2013), an additional mechanism by which CBD may decrease the firing discharge observed in our study. In sharp contrast to the aforementioned observations, we also documented CBD’s lack of modulatory actions on the outward macroscopic K^+^ currents, suggesting that CBD does not interact with the transient fast-inactivating or delayed K^+^ current channels mediating *I*_A_ and *I*_M_, respectively. Despite this caveat, the modulatory actions of CBD on the different somatic K^+^ channel responses are consistent with a mechanism that favors a decreased output discharge of human pyramidal neurons.

Notably, our study confirmed the modulatory action of CBD on the voltage-sensitive Na^+^ currents underlying spike generation in human pyramidal neurons. Previous studies in animal models have demonstrated that the interaction between CBD and Na^+^ channels results in decreased AP discharge and decreased excitability in brain regions critical for seizure propagation (Kaplan et al., 2017; Khan et al., 2018). Notably, CBD decreases the firing discharge of pyramidal neurons but increases the output of parvalbumin-positive GABAergic interneurons (Kaplan et al., 2017; Khan et al., 2018). The dual action of CBD on neuronal output may reflect a differential CBD-mediated coupling to intracellular signaling mechanisms specific to glutamatergic and GABAergic neurons. This coupling may involve distinct ion channels, neurotransmitter receptors, and intracellular signaling cascades. Although appealing, this possibility requires additional investigation beyond our study’s scope.

Lastly, we acknowledge the limitations of this study, including the heterogeneity in the origin of the neocortical human neurons because of the scarce availability of human brain tissue and the relatively small study sample. Although our findings may be considered modest, they provide direct evidence of CBD’s modulatory actions on the electrophysiological parameters underlying the neuronal hyperexcitability associated with epilepsy. More importantly, our results reinforce clinical observations reporting CBD’s positive effects in treating patients with DRE.

## Materials and methods

### Human coronal slice preparation

Neocortical brain tissue of the parietal, the frontal, and the temporal neocortex was obtained during surgical procedures of nine patients with DRE (see Supplementary Table S1 for clinical data). All individuals underwent a thorough presurgical evaluation, which included electroencephalography and magnetic resonance imaging. The study protocol (055/2018) received approval from the scientific and ethics committees of the participating institutions. All participants provided written informed consent before they were included in the study. After resection, brain tissue from the epileptic focus was placed in an ice-cold isotonic buffer solution (in mM: 320 sucrose, 1 EDTA, 5 Tris-HCl; pH = 7.4 and 300–310 mOsm), with continuous bubbling of carbogen (95% O_2_/5% CO_2_ at 0.5 L/min), and transported to the neurophysiology laboratory (<45 min). In the laboratory, brain tissue samples were sliced perpendicular to the pial surface using a vibratome (Leica VT1000S; Nussloch, Germany) in the presence of an iced sucrose solution (with continuous carbogen bubbling), with the following composition (in mM): 210 sucrose, 2.8 KCl, 2 MgSO_4_, 1.25 Na_2_HPO_4_, 25 NaHCO_3_, 1 MgCl_2_, 1 CaCl_2_, and 10 D-glucose; pH ≈ 7.30–7.35 and 300–310 mOsm. Next, the resulting coronal slices (350-µM thickness) were maintained at 34 C for 25–30 min in an artificial cerebrospinal fluid (ACSF) solution (pH ≈ 7.30–7.35) with the following composition (in mM): 125 NaCl, 2.5 KCl, 1.25 Na_2_HPO_4_, 25 NaHCO_3_, 4 MgCl_2_, 1 CaCl_2_ and 10 D-glucose; 300–310 mOsm. Lastly, the slices were maintained at room temperature for at least 1 h before the electrophysiological recordings were performed. For the recordings, the ACSF contained (in mM): 125 NaCl, 2.5 KCl, 1.25 Na_2_HPO_4_, 25 NaHCO_3_, 1.5 MgCl_2,_ 2.5 CaCl_2_, and 10 D-glucose; pH ≈ 7.30–7.35 and 300–310 mOsm. The ACSF was continuously perfused to the slices as an extracellular solution at a flow rate of ∼2 mL × min^-1^. The recordings were performed at 32.0°C ± 1°C. We restricted data collection to one train stimulation (extracellular recording) or one neuron (whole-cell patch clamp) per slice. For each patient, we analyzed no more than two slices, thereby reducing within-subject bias, emphasized cross-subject comparisons and ensuring a more representative sampling across individuals.

### Extracellular recordings

A bipolar stimulation electrode was placed in layer I/II, and the resulting field excitatory postsynaptic potential (fEPSP) was recorded in the dendritic region of layer V with a borosilicate pipette (one to two MΩ when filled with 3M NaCl). The current stimuli were delivered via a high-voltage isolation unit (A365D; World Precision Instruments, Sarasota, FL, United States) under the control of a Master-8 pulse generator (AMPI, Jerusalem, Israel). The electrical responses were amplified with a Dagan BVC-700A device (Minneapolis, MN, United States) connected with a 100x-gain unit (Dagan, model 8,024) and a high-pass filter set at 0.3 Hz. Additional noise suppression was accomplished with a Hum Bug Noise Eliminator (Quest Scientific Instruments; North Vancouver, BC, Canada). The evoked fEPSPs were digitized with an A/D converter (BNC-2110) for storage and offline analysis with custom-made software written for LabVIEW 7.1 (National Instruments, Austin, TX, United States).

After a stabilization period in the recording chamber (15–20 min), a baseline of fEPSPs was acquired at 0.067 Hz for 15 min (using current pulses of 200–250 μA, 100-µs duration) followed by perfusion of CBD (15 min, 10 µM) or its vehicle (DMSO, 0.05%) and subsequent drug washout to examine changes in the strength of synaptic transmission. The effect of CBD was quantified during the last 3 min of drug perfusion and compared with the baseline response. In another set of experiments, 4-AP (200 µM) was bath perfused, and the number and frequency (Hz) of spontaneous events in response to a single current pulse (350–450 μA, 100 µs) were quantified in a 500-ms period (from 50 ms post-stimulus to 550 ms post-stimulus). Then, CBD was perfused to determine its effect on spontaneous activity.

### Whole-cell patch-clamp recordings

The whole-cell patch-clamp recordings were restricted to pyramidal-like cells located in layer V. The neurons were visualized using DIC infrared illumination coupled to an FN1 eclipse microscope (Nikon Corporation, Tokyo, Japan). The patch pipettes were pulled from borosilicate glass using a micropipette puller (P97, Sutter Instruments, Novato, CA, United States). The pipette tips had a resistance of 3–5 MΩ when filled with an intracellular solution (pH ≈ 7.20–7.28) with the following composition (in mM): 135 K^+^-gluconate, 10 KCl, 5 NaCl, 1 EGTA, 10 HEPES, 2 Mg^2+^-ATP, 0.4 Na^+^-GTP, and 10 phosphocreatine; 295 mOsm. Whole-cell recordings were performed using an Axopatch 200B amplifier (Molecular Devices, San José, CA, United States), digitized at 10 kHz, and filtered at 2 kHz with a Digidata 1550B (Axon Instruments, Palo Alto, CA, United States). Digital signals were acquired and analyzed offline with Clampfit 11.3 software (Molecular Devices). Seal quality was tracked online, incorporating the following criteria: access resistance <15 MΩ with <10% drift), holding currents <100 pA, and a stable resting membrane potential.

### Determination of passive and active electrophysiological properties

A series of 1-s current pulses (from −300 to 90 pA; Δ30 pA) were injected to determine the current–voltage (I–V) relationship, the somatic input resistance, and the membrane time constant. The input resistance was determined as the slope of a linear fit ( $\left.f\left(x\right)=mx+b\right)$ to the steady-state I–V plot elicited by subthreshold current injection (−300 to 0 pA). The time constant was calculated by fitting a single exponential function $\left(f\left(t\right)=\sum_{i=1}^{n}A_{i}e^{‐\frac{t}{\tau i}}+C\right)$ to a voltage response elicited by a current pulse of −30 pA. The membrane capacitance (C_m_) was computed by the ratio of the time constant to the input resistance of the cell. To quantify the sag amplitude, the difference between the initial peak hyperpolarization and the steady-state membrane potential was measured during the −300-pA hyperpolarizing current step. The rheobase was determined via somatic injection of current ramps ranging from −100 pA to the threshold for spike generation, with increments of 30 pA and a duration of 500 ms. We employed this variable to evaluate the intrinsic excitability of cortical pyramidal neurons.

### Spike dynamics and firing analysis

All spike kinetic measurements were computed from the AP spikes elicited with current injections (200 pA). Spikes from individual trains were averaged to compute the first derivative of the membrane potential, dV/dt (mV/ms), which was then plotted as a function of the membrane potential to generate phase plots. To assess the firing output (f_(I)_) of pyramidal neurons, a series of firing rate–current curves were constructed by computing the number of elicited spikes in response to current injections from 0 to 390 pA. The output gain was determined with a three-parameter sigmoid function, $f_{\left(I\right)}=a/\left(1+e^{-k\left(I-I0\;\right)}\right)$ , where f_(I)_ corresponds to the firing rate as a function of the input current (I), and k defines the output gain. The offset was estimated as the x-intercept from a linear projection of the straight part of the plot.

### Macroscopic currents

Voltage-dependent ion currents were elicited with 10-mV incremental steps from −100 to +50 mV (500 ms), with a holding potential set at −70 mV in voltage-clamp mode. The P/N leak subtraction protocol was applied online to exclude unwanted capacitive transients and leak current components irrelevant to the macroscopic ionic currents. This method subtracts linear, voltage-independent capacitive, and leak currents from the recorded signal, ensuring that only the ionic currents remain for analysis. The current density was calculated by normalizing the (inward or outward) peak or the (outward) steady-state current values to the total membrane surface area, estimated from the whole-cell capacitance. To examine the recovery from fast inactivation, we used a multiple-pulse protocol executed with a double conditioning depolarization from a holding potential of −70 to 0 mV (5 ms); then, a subsequent test pulse at the equal depolarizing pulse value (0 mV) was delivered beforehand to evoke the inward current fraction. The recovery rate was determined by the time between conditioning and test pulses, and probe pulses were increased from 5 to 75 ms in 5-ms augmentations. The recovery time constant from inactivation was obtained by plotting the ratio I_test_/I_conditioning_ against the interval time (Δt), and the data were fitted with a single exponential equation.

## Materials

All drugs and chemicals used in this study were purchased from Sigma Aldrich Chemical Co. (St. Louis, MO, United States). TTX was obtained from Alomone Labs (Jerusalem, Israel). Cannabidiol was obtained from Hemp Meds, LLC, United States . The CBD concentration used in this study was previously determined based on a logarithmic dose-response curve of glutamate release inhibition from human cortical synaptosomes (Martínez-Aguirre et al., 2023). Drugs were constantly dissolved as a stock and then diluted into ACSF at their final concentration and employed on the same experimental day.

### Statistical analysis

Results are given as mean ± SEM; n refers to the number of cells from which recordings were made. Clampfit 10.7 (Molecular Devices, United States) and GraphPad Prism 8 software (GraphPad, United States) were employed for data analysis, curve fitting, and plotting. Prior to statistical comparison, data distribution was assessed using the Shapiro–Wilk test to confirm normality. Student’s paired t-test was employed for comparing two groups; the threshold for statistical significance was established at p < 0.05 for all analyses. Responsiveness to CBD was defined based on comparisons of pre- and post-perfusion values; the changes exceeding ±2 SEM were used as the threshold to define responsiveness of slices (extracellular recordings) or neurons (whole cell experiments).

## Data Availability

The raw data supporting the conclusions of this article will be made available by the authors, without undue reservation.
