# Supplementary material for: Cannabidiol reduces synaptic strength and neuronal firing in layer V pyramidal neurons of the human cortex with drug-resistant epilepsy
Source: Front Pharmacol. 2025 Jul 22;16:1627465. doi: 10.3389/fphar.2025.1627465 (PMC12321821; doi:10.3389/fphar.2025.1627465)
Supplement: Supplementary file 1 [file Table1.docx]

| **Surgery date** | **Sex** | **Age**  **(years)** | **Seizure onset (years)** | **Duration**  **(years)** | **Seizure frequency**  **(per month)** | **Location of epileptic focus** |
| --- | --- | --- | --- | --- | --- | --- |
| July, 22 | Female | 21 | 10 | 11 | 7 | Temporal neocortex |
| August, 22 | Male | 21 | 13 | 8 | 12 | Temporal neocortex |
| July, 22 | Female | 30 | 13 | 17 | 20 | Frontal neocortex |
| January, 23 | Female | 27 | NA | NA | NA | Temporal neocortex |
| March, 23 | Male | 16 | 10 | 6 | 150 | Frontal neocortex |
| May, 23 | Female | 24 | 16 | 8 | NA | Frontal neocortex |

**Table 1.** Clinical data of neocortical tissue resected from patients with drug-resistant epilepsy.
